# Use of artificial intelligence for reverse referral between a hospital emergency department and a primary urgent care center

**DOI:** 10.3389/fdgth.2025.1546467

**Published:** 2025-03-03

**Authors:** Yolanda Taules, Silvia Gros, Maria Viladrosa, Natàlia Llorens, Sílvia Solis, Oriol Yuguero

**Affiliations:** ^1^ Emergency Department, University Hospital Arnau de Vilanova, Lleida, Spain; ^2^ ERLaB, Grupo de Investigación en Urgencias, IRBLLEIDA, Lleida, Spain; ^3^ Primary Care Emergency Center, Catalan Health Instiute (ICS), Lleida, Spain, 4eHealth Center, Universitat Oberta de Catalunya (UOC), Barcelona, Spain; ^4^ eHealth Center, Universitat Oberta de Catalunya, Barcelona, Spain

**Keywords:** AI, emergency care, primary care, health serivces, medical informatic applications

## Abstract

**Background:**

The demand for immediate care in emergency departments (EDs) has risen since the severe acute respiratory syndrome coronavirus 2 (SARS-CoV-2) pandemic.

**Objective:**

Test the ability of AI to promote reverse referral and to provide patient education.

**Methods:**

Pilot study that included patients presenting to our Hospital Emergency Department (HED) with a non severe disease and who met the inclusion criteria. The participants were asked to answer a series of questions using an electronic device and receive a recommendation for health attention. Then, patients could choose to either remain in the hospital or leave.

**Results:**

427 patients finally participated in the pilot study. Within this population, 49.5% were women, and the mean patient age was 37.5 years. Mediktor recommended reverse referral to urgent care in 43.6%. Our results demonstrate that the tool is safe and provides accurate patient screening, correctly distinguishing between those who should continue to wait for HED care and those for whom an urgent care center is adequate.

## Introduction

Patients who seek emergency or urgent care have varying levels of complexity, from life-threatening emergencies to minor or non-urgent conditions. The demand for immediate care in emergency departments (EDs) has risen since the severe acute respiratory syndrome coronavirus 2 (SARS-CoV-2) pandemic. In our facility, ED visits by patients with non-severe conditions increased significantly in 2022 and 2023, returning to 2019 levels (62.1%) (1).

When a patient arrives at a hospital emergency department (HED), nursing staff conduct structured triage to prioritize care according to the urgency of their condition (2) and optimize wait times. According to this system, patients requiring resuscitation are assigned urgency level 1, whereas those with non-urgent care needs are classified as level 5. Currently, all HEDs in the public network of the Catalan Health System have a system of structured triage (Triage Model of Andorra, version 4.5).

Given the number of non-severe patients who seek care in HEDs, we set out to establish a model of reverse referral (3). Under this model, patients identified during triage as having non-severe or non-emergent conditions are redirected to urgent care facilities outside the hospital. This initiative aligns with the Catalonia National Emergency Care Plan (PLANUC) (4), which encourages care delivery in the most appropriate facility to ensure effective and timely services.

A 2017 study published in Emergencias (5) showed that Mediktor®, an artificial intelligence (AI) symptom-assessment tool, provides useful diagnostic support and could improve ED efficiency as an adjunct to conventional triage (6).

AI is not a novel concept. It was first defined by the physicist John McCarthy in the 1950s as “(...) the science and engineering of making intelligent machines, especially intelligent computer programs” (7). Although alternative definitions have emerged in recent years, here we adopt the definition of the European Commission, that is, “software (and possibly also hardware) systems designed by humans that, given a complex goal, act in the physical or digital dimension by perceiving their environment through data acquisition, interpreting the collected structured or unstructured data, reasoning on the knowledge, or processing the information, derived from this data and deciding the best action(s) to take to achieve the given goal” (8).

Given the current situation in EDs worldwide, some argue that AI could improve flows and strengthen the management of these departments, which face chronic overcrowding.

A study carried out in Spain in 2024 (9) gave an overview of the different applications of AI in EDs: self-triage, in which patients receive symptom-assessment support before visiting the ED; improving care delivery; and improving information management. Indeed, a recent report (10) found that AI tools outperformed physicians in tasks such as interpreting radiographs or diagnosing certain diseases based on visual evidence. However, clinical expertise must be a basic component when processing recommendations from technological sources.

Although the safety of reverse referral is supported by published evidence, certain hospitals are reluctant to adopt the procedure (11). Given that previous research has found that reverse referral leads to a reduction in ED visits with no increase in mortality, our aims in this study were to test the ability of AI to promote reverse referral and to provide patient education.

## Materials and methods

### Test design

We carried out a pilot study that included patients presenting to our HED with a condition classified as level IV or V according to the Andorran triage system and who met the inclusion criteria. The participants were asked to answer a series of questions using an electronic device.

At the conclusion of each assessment, the application recommended that the patient either continue waiting in the HED or go to an urgent care center located 10 min away from our hospital on foot.

At this point, patients could choose to either remain in the hospital or leave. We simultaneously developed a digital tool that enabled patients referred to the urgent care center to be given the most prompt care possible on arrival.

### Mediktor technology

Mediktor's system integrates Artificial Intelligence, specifically Natural Language Processing (NLP), Machine Learning, and Bayesian networks, with a proprietary medical database to provide a recommendation.

#### Natural language processing (NLP)

NLP is an artificial intelligence technology that enables computers to understand, interpret, and generate human language. Mediktor utilizes NLP at the outset of its evaluation to comprehend a user's symptoms and signs, employing a proprietary algorithm designed to recognize multiple symptoms and signs simultaneously.

#### Learning patterns

The model is trained to identify patterns and relationships within medical data, enabling it to determine the most appropriate subsequent question based on the specific characteristics of the case and previously learned patterns from similar cases. Mediktor utilizes machine learning techniques such as neural networks and Bayesian networks to train its artificial intelligence model.

• **Machine Learning** Machine learning is an artificial intelligence technique used to train computational models and systems to perform specific tasks without explicit programming. By training the AI with vast amounts of medical data, it can identify patterns and relationships between variables such as symptoms, signs, risk factors, and more.
• **Bayesian Networks** Bayesian networks are probabilistic graphical models used to represent and reason about uncertain situations. In other words, these networks aid in making predictions and decisions based on probabilities. The networks represent relationships between variables in a graph, where each variable is represented as a node, and the relationships between them are represented by edges. Once the graph has been constructed, the network can utilize probabilities to make inferences for more accurate and effective decision-making.

#### Medical database

Mediktor's artificial intelligence relies on a proprietary medical database, a structured repository of medical information. It contains clinical data such as symptoms, signs, diseases, medical and family history, encoded using standardized medical terminology for consistent interpretation. The database is continuously updated with the latest medical advancements.

This data serves as a crucial resource for the artificial intelligence to correlate symptoms with potential preliminary diagnoses, urgency levels, and recommended care.

### Exclusion criteria

Patients were excluded from the pilot study if they were younger than 18 or older than 65 years or if they met any of the criteria appearing in Table 1.

**Table 1:** Exclusion criteria for Mediktor proposals.

Patients with delirium or Glasgow coma scale scores <15
Pregnant women
Patients presenting with gynecological/obstetric diseases
Patients unable to travel to the urgent care center by their own means
Patients residing outside Catalonia
Patients with a history of gynecological disease
Patients with polytrauma
Patients who are uncooperative or may cause disruptions in the protocol

### Statistical analysis

This was a descriptive study with consecutive patient inclusion. The statistical analyses included the chi-square test, the Levene test, and the Game-Howell test depending on the variables analyzed. We analyzed the sociodemographic characteristics of the participants, the triage level assigned, the proposal given by Mediktor, and the final decision made by the patient. Two weeks after the index visit, we determined whether the patients had gone to the urgent care center or not, the care delivered in both the hospital and in urgent care, and recorded any return HED visits. Lastly, patient satisfaction was evaluated.

### Ethical considerations

The study was approved by the Universitat Oberta de Catalunya Research Ethics Committee (approval number, CE23-PR31). Informed consent was obtained from all participants before they participated in the study. All study methods were applied in accordance with the appropriate guidelines and regulations. All processing, communication, and transfer of participant personal data was performed in compliance with Organic Law 3/2018, of December 5, on personal data protection and guarantee of digital rights (LOPD-GDD 3/2018) and Regulation (EU) 2016/679 of the European Parliament and of the Council, of April 27, 2016.

## Results

Between November 6, 2023, and March 1, 2024, 6,762 patients visited our HED and were assigned a triage level of IV or V. The study sample for our test included 462 patients recommended for reverse referral, representing 6.8% of the total.

Of these, we obtained data for the different study variables for 427 patients. Within this population, 49.5% were women, and the mean patient age was 37.5 years. A triage level of IV was assigned to 82.2% of the patients included, and Mediktor recommended reverse referral to urgent care in 43.6%.

The correlation between the Mediktor recommendations and patient decisions is shown in Table 2. In total, 84.1% of patients presented to the urgent care center, including those recommended to do so as well as patients advised to remain in the hospital (*P* < 0.001). The Fisher's exact test revealed a statistically significant association (*p* = 0.001) between the Mediktor proposal and the patient's decision However, no significant association was found between triage level and the Mediktor proposal. (*p* = 0,487).

**Table 2:** Patient decisions after receiving the mediktor recommendation.

Mediktor proposal	Patient decision	
	Primary urgent care center	Hospital Universitari Arnau de Vilanova
Primary urgent care center	169 (90.9%)	17 (9.1%)
Hospital Universitari Arnau de Vilanova	190 (78.8%)	51 (21.2%)
Total	359 (84.1%)	68 (15.9%)

As shown in Table 3, 25.9% of the participants ultimately sought no care in either type of facility.

**Table 3:** Patients presenting to an urgent care center following assessment.

Mediktor proposal	Patient decision		
	Hospital Universitari Arnau de Vilanova	Primary urgent care center	Home
Primary urgent care center	17 (9.10%)	136 (73%)	33 (17.20%)
Hospital Universitari Arnau de Vilanova	51 (21.20%)	112 (59%)	78 (19.30%)
Total	68 (15.9%)	248 (58.07%)	111 (25.9%)

When Mediktor recommended going to an urgent care center, 78% of the patients who followed this recommendation required no testing or treatment. Within 2 weeks of the index visit, 16.1% of the patients visited the hospital again, although none required admission.

Of those patients who opted to wait in the hospital despite the recommendation to seek urgent care, 46% required additional testing. All except 1 patient were discharged, and 6.7% went for a return ED visit within 2 weeks of the index visit.

None of the 51 patients who followed the recommendation to remain in the hospital were admitted. However, 54% required diagnostic testing and 23% received intravenous or intramuscular treatment. There was a 24% rate of return ED visits following the index visit.

No significant differences were observed in patient adherence to the Mediktor recommendations based on educational level or economic status. However, differences were found in satisfaction with reverse referral and with the application.

Data regarding patient satisfaction are shown in Table 4. Patients with secondary education were more satisfied with the Mediktor application than the other groups (*P* = 0.037)*.* The overall rate of patient satisfaction was 7.82/10 for the tool and 7.74/10 for the reverse referral procedure.

**Table 4:** Participant satisfaction by educational level.

Educational level	Satisfaction with reverse referral			Satisfaction with Mediktor		
	Mean	SD	*P*	Mean	SD	*P*
Primary	7.83	1.89	0.736	7.83	1.73	0.037*
Secondary	7.75	2.19		8.09	2.04	
Vocational training	7.63	2.4		7.49	2.48	
High school graduate	7.73	2.2		7.9	2.38	
University	7.77	2.46		7.86	1.66	
	7.74	2.211		7.91	2.1	

* Statistical significance.

## Discussion

The findings from this study demonstrate that the Mediktor tool is useful to the reverse referral process and well-received by patients and healthcare professionals. Nearly 7% of patients were included in the group recommended for reverse referral. Reverse referral is a common practice in Spain and is increasingly being adopted in other countries. In Scotland, a similar procedure was instituted safely and was met with a high rate of patient acceptance (12).

Despite the safety of the procedure, other reports have observed difficulties that hamper the proper functioning of the system (13), which led to the development of the AI tool evaluated in this study.

The results of this study reveal that the AI tool is safe. The patients encouraged to remain in the hospital required more diagnostic testing and/or intravenous treatments. These individuals also had a higher rate of return visits. Of the patients recommended to go to the urgent care center by the tool, 78% required no testing or treatment, and their rate of return ED visits was lower. These findings confirm that the digital tool is effective in triaging non-severe conditions. The screening performed by the tool was accurate, as patients advised to remain in the hospital required more diagnostic tests and treatments. A review published in 2020 (14) evaluated a series of symptom checkers, which likely served as a foundation for the tool evaluated in this study.

It is important to note that many patients opted to seek medical attention at an urgent primary care facility despite being recommended to remain in the hospital. While no statistically significant associations were found between sociodemographic factors such as education and socioeconomic status and adherence to recommendations, our analysis revealed that patients who arrived at the Emergency Department via public transportation or on foot were more likely to follow the recommendation to seek care at a Primary Care Urgent Care Center. Conversely, patients who relied on private transportation were less likely to do so. Notably, despite the tool's recommendation to remain in the hospital, a significant proportion of patients chose to seek care at the primary care center, often citing a lack of awareness of this alternative.

In addition, patients reported highly favorable perceptions of primary care delivery, suggesting that these care levels should be given greater priority. The results concerning satisfaction with the reverse referral process and the tool were unexpected. Our findings indicate that patients with lower levels of education expressed greater satisfaction with being directed to the Primary Care Urgent Care Center. The majority of these patients were previously unaware of this service and reported being satisfied with the care provided for non-urgent conditions.

Our findings suggest that patients with lower levels of education (primary and secondary) may have derived greater benefit from the digital tool. The tool's user-friendly interface, characterized by its use of visual aids and plain language, may have been particularly appealing to this population, who may have had limited prior experience with health technologies. The use of colloquial language to describe signs and symptoms was consistently highlighted as a positive feature by these patients.

We believe that this study achieved its objective of raising patient awareness regarding the severity of the symptoms that lead them to visit the hospital. It is significant that 25% of the patients who presented to the HED of their catchment area eventually returned home without consulting a healthcare provider. This finding underscores the potentially inappropriate use of ED resources, confirming a widely held belief among healthcare professionals (15). Tools such as Mediktor could play a role in addressing this issue by assisting with patient education. Furthermore, with the upcoming introduction of emergency medicine as a recognized specialty in Spain (16), it will be essential to incorporate AI tools in residency training, as these will become part of daily ED workflows.

Given that this is a pilot study exploring the utility of this tool, several limitations inherent to this type of research must be acknowledged.

The primary limitation of this study is its descriptive design, which precluded proper comparison between patients managed with and without the AI tool. In fact the use of this study design restricts the ability to infer causality or robustly compare the outcomes of AI-assisted triage vs. standard practices. Furthermore, there is a limitation regarding the sample size (*n* = 427), which could restrict the sample's representativeness. Exclusion of elderly and pediatric patients may limit the generalizability of the results. The risk of misclassifying patients (e.g., recommending urgent care for those requiring hospital services) is a critical limitation, and with this study we haven't seen long-term implications. Due to the exploratory nature of this pilot study, precisely quantifying false positives and false negatives is difficult. It is important to note that false negatives in this context refer to patients who were incorrectly advised to seek urgent care. Given the tool's conservative approach, the rate of false positives is likely higher, as patients may have been unnecessarily retained in the hospital when they could have safely been discharged to primary care.

In conclusion, our results demonstrate that the tool is safe and provides accurate patient screening, correctly distinguishing between those who should continue to wait for HED care and those for whom an urgent care center is adequate. Given the high patient satisfaction with the procedure, it is important to raise awareness of the various urgent and emergency care resources available, especially those at the primary level. This study employed descriptive statistics. Nevertheless, the use of advanced modeling techniques, including logistic regression and machine learning, is essential to more precisely identify predictors of adherence to or success of recommendations.

Future research could explore the potential of home self-triage tools, which could provide patients with appropriate recommendations before they visit the hospital.

## Data Availability

The original contributions presented in the study are included in the article/Supplementary Material, further inquiries can be directed to the corresponding author.

## References

[1] Informe breu núm. 41: Activitat d’urgències. Catalunya 2019. Barcelona. Catsalut. Document in Catalan. Servei Català de la Salut. 2021. Available online at: https://catsalut.gencat.cat/ca/detalls/noticies/2020-11-12-informe-breu-41-activitat-urgencies-201 (accessed May 12, 2024)

[2] Ovens H. Emergency department overcrowding. A proposal for the system from within the system. Emergencias. (2010) 22:244–6.

[3] Álvarez C Vázquez MJ. Relation between emergency department caseload and transfers from regional to other hospitals. Emergencias. (2010) 22:28–32.

[4] Urgent care in times of COVID-19. 2020. Consell assessor del Pla Nacional d’urgències (PLANUC). Document in Catalan. Available online at: https://canalsalut.gencat.cat/web/content/_A-Z/C/coronavirus-2019-ncov/materialdivulgatiu/document-sobre-atencio-urgent-temps-covid-19.pdf (accessed June 18, 2021)

[5] Moreno E Pueyo I Sánchez M Martín M Masip J. A new artificial intelligence tool for assessing symptoms in patients seeking emergency department care: the mediktor application. Emergencias. (2017) 29:391–6. Article in Spanish. 29188913

[6] Soler W Gómez M Bragulat E Álvarez A. Triage: a key tool in emergency care. An Sist Sanit Navar. (2010) 33:55–68. Article in Spanish. 10.4321/S1137-66272010000200008 20508678

[7] Artificial Intelligence definitions. Human-Centered artificial Intelligence. Stanford University. Available online at: https://hai.stanford.edu/sites/default/files/2020-09/AI-Definitions-HAI.pdf (accessed November 14, 2023)

[8] European Commission. European AI Alliance. Available online at: https://ec.europa.eu/futurium/en/european-ai-alliance/ecs-definition-ai-or-how-define-artificial-intelli-gence-real-and-concerned.html (accessed November 14, 2023)

[9] Castro-Delgado R Pardo Ríos M. Artificial intelligence and emergency services: we need to take a step forward. Emergencias. (2024) 36:145–7. 38597621 10.55633/s3me/002.2024

[10] Vearrier L Derse AR Basford JB Larkin GL Moskop JC. Artificial intelligence in emergency medicine: benefits, risks, and recommendations. J Emerg Med. (2022) 62(4):492–9. 10.1016/j.jemermed.2022.01.001 35164977

[11] Leey-Echavarría C Zorrilla-Riveiro J Arnau A Fernàndez-Puigbó M Sala-Barcons E Gené E. Model to predict risk for hospital admission and indicate the safety of reverse triage in a hospital emergency department: a prospective validation study. Emergencias. (2022) 34:165–73. 35736520

[12] Bentley JA Thakore S Morrison W Wang W. Emergency department redirection to primary care: a prospective evaluation of practice. Scott Med J. (2017) 62(1):2–10. 10.1177/0036933017691675 28173740

[13] Morin C Choukroun J Callahan JC. Safety and efficiency of a redirection procedure toward an out of hours general practice before admission to an emergency department, an observational study. BMC Emerg Med. (2018) 18(1):26. 10.1186/s12873-018-0173-6 30134934 PMC6103978

[14] Schmieding ML Kopka M Schmidt K Schulz-Niethammer S Balzer F Feufel MA. Triage Accuracy of symptom checker apps: 5-year follow-up evaluation. J Med Internet Res. (2022) 24(5):e31810. 10.2196/31810 35536633 PMC9131144

[15] Fernández Alonso C Aguilar Mulet JM Romero Pareja R Rivas García A Fuentes Ferrer ME González Armengol J Hiperfrecuentación en atención Primaria e hiperfrecuentadores en urgencias [frequent attenders in primary health care centres and frequent attenders in emergency departments]. Atencion primaria. (2018) 50(4):222–7. 10.1016/j.aprim.2017.02.011 28610846 PMC6837134

[16] Draft Royal Decree establishing the specialty of emergency medicine and updating certain aspects related to the training of specialists in family and community medicine. Document in Spanish. Available online at: https://www.sanidad.gob.es/normativa/audiencia/docs/DG_45_23_PRD_Titulo_especialista_Medicina_Urgencias_y_Emergencias.pdf (accessed December 15, 2023)

